# Semaglutide Ameliorates Diabetic Neuropathic Pain by Inhibiting Neuroinflammation in the Spinal Cord

**DOI:** 10.3390/cells13221857

**Published:** 2024-11-08

**Authors:** Sing-Ong Lee, Yaswanth Kuthati, Wei-Hsiu Huang, Chih-Shung Wong

**Affiliations:** 1 Department of Anesthesiology, Cathay General Hospital, Taipei 106, Taiwan; onguitar79@gmail.com (S.-O.L.); yaswanthk1987@gmail.com (Y.K.); cgh17178@cgh.org.tw (W.-H.H.); 2 Department of Health and Leisure Management, Yuanpei University of Medical Technology, Hsinchu City 306, Taiwan; 3 Graduate Institute of Medical Sciences, National Defense Medical Center, Taipei 114, Taiwan

**Keywords:** diabetic neuropathic pain, GLP-1RA, SEMA, microglia, astrocytes, pro-inflammatory cytokines

## Abstract

Glucagon-like peptide 1 (GLP-1) receptor agonists are frequently used to treat type 2 diabetes and obesity. Despite the development of several drugs for neuropathic pain management, their poor efficacy, tolerance, addiction potential, and side effects limit their usage. Teneligliptin, a DPP-4 inhibitor, has been shown to reduce spinal astrocyte activation and neuropathic pain caused by partial sciatic nerve transection. Additionally, we showed its capacity to improve the analgesic effects of morphine and reduce analgesic tolerance. Recent studies indicate that GLP-1 synthesized in the brain activates GLP-1 receptor signaling pathways, essential for neuroprotection and anti-inflammatory effects. Multiple in vitro and in vivo studies using preclinical models of neurodegenerative disorders have shown the anti-inflammatory properties associated with glucagon-like peptide-1 receptor (GLP-1R) activation. This study aimed to investigate the mechanism of antinociception and the effects of the GLP-1 agonist semaglutide (SEMA) on diabetic neuropathic pain in diabetic rats. Methods: Male *Wistar* rats, each weighing between 300 and 350 g, were categorized into four groups: one non-diabetic sham group and three diabetic groups. The diabetic group received a single intraperitoneal injection of streptozotocin (STZ) at a dosage of 60 mg/kg to induce diabetic neuropathy. After 4 weeks of STZ injection, one diabetic group was given saline (vehicle), and the other two were treated with either 1× SEMA (1.44 mg/kg, orally) or 2× SEMA (2.88 mg/kg, orally). Following a 4-week course of oral drug treatment, behavioral, biochemical, and immunohistochemical analyses were carried out. The mechanical allodynia, thermal hyperalgesia, blood glucose, advanced glycation end products (AGEs), plasma HbA1C, and spinal inflammatory markers were evaluated. Results: SEMA treatment significantly reduced both allodynia and hyperalgesia in the diabetic group. SEMA therapy had a limited impact on body weight restoration and blood glucose reduction. In diabetic rats, SEMA lowered the amounts of pro-inflammatory cytokines in the spinal cord and dorsal horn. It also lowered the activation of microglia and astrocytes in the dorsal horn. SEMA significantly reduced HbA1c and AGE levels in diabetic rats compared to the sham control group. Conclusions: These results indicate SEMA’s neuroprotective benefits against diabetic neuropathic pain, most likely by reducing inflammation and oxidative stress by inhibiting astrocyte and microglial activity. Our findings suggest that we can repurpose GLP-1 agonists as potent anti-hyperalgesic and anti-inflammatory drugs to treat neuropathic pain without serious side effects.

## 1. Introduction

Neuropathic pain (NP) is defined as pain resulting from damage or injury to the somatosensory system [1]. This pain is challenging to manage, with common causes including chemotherapy, spinal cord injury, diabetes, ischemic illness, HIV, alcoholism, multiple sclerosis, and surgical procedures [2]. Diabetic neuropathic pain (DNP) represents a type of nerve damage that often develops in individuals diagnosed with diabetes. Elevated blood glucose levels can harm nerves throughout the body, predominantly affecting those in the legs and feet. DNP is primarily linked to symptoms such as allodynia, characterized by pain triggered by normally non-painful stimuli, and hyperalgesia, which refers to increased sensitivity to pain [3]. DNP impacts the quality of life by disrupting mood, sleep, libido, mobility, employment, and leisure and social activities [3]. First-line treatments for neuropathic pain management include tricyclic antidepressants and serotonin-noradrenaline reuptake inhibitors, along with anticonvulsants like pregabalin and gabapentin. Second-line medications consist of tramadol or topical lidocaine, while opioids such as morphine serve as third-line options [3].

Recent preclinical and clinical research findings have questioned the appropriate application of opioid analgesics in neuropathic pain management [4]. While morphine and other opioids continue to be the “gold standard” for managing acute pain, their effectiveness in treating chronic pain syndromes like diabetic neuropathy, a common complication in diabetic patients, seems to be limited [4]. A decrease in the quantity of opioid receptors or a disruption in µ-opioid receptor–G protein coupling reduces the antinociceptive efficacy of morphine in preclinical and clinical models of diabetic neuropathic pain (DNP) [5,6,7,8]. In a similar manner, the spinal dorsal horn of animals exhibiting DNP demonstrates a significantly decreased density of opioid receptors [9]. In preclinical animal models, it is known that variations in blood or brain glucose concentrations affect baseline nociceptive processing and opioid antinociception [10,11]. Additionally, some research indicates that DM may have a direct impact on the brain’s production and concentration of beta-endorphins, which may change the morphine response in the DNP rats [12]. Furthermore, it has been observed that morphine’s antinociception is attenuated in the CNS of the DNP rats due to a notable increase in L-type Ca^2+^ channels [13]. Our findings, along with other research reports, suggest that morphine’s antinociceptive effect in the DNP rats was diminished [14,15].

Thus, there is a need for safe analgesics with better tolerance for the management of DNP. Nevertheless, the discovery of a new drug is an expensive and long process with an estimated cost of up to $3 billion and an average time of 15 years for one drug. It is estimated that more than 90% of the candidate drug compounds failed to enter the market due to a lack of safety or efficiency [16]. Hence, there is a need to explore whether any of the currently approved drugs can be repurposed for the treatment of DNP. Drug repurposing involves identifying new applications for existing marketed drugs and is regarded as a highly effective strategy in the realm of drug discovery. Repurposed pharmaceuticals minimize the time and expense associated with discovering novel therapies, as pharmacokinetic and toxicological data for existing medications are readily accessible. A variety of drugs like antineoplastics, anticonvulsants, antidepressants, antibiotics, antimalarial, and antihypertensive drugs have been repositioned as analgesics for pain management with FDA approval [17]. However, very few of them are approved for DNP management.

Our laboratory has previously investigated the analgesic efficacy of the DPP-4 inhibitor teneligliptin in an animal model of neuropathic pain involving the partial transection of the sciatic nerve [18]. Subsequently, we have established the effectiveness of teneligliptin in mitigating morphine tolerance in a DNP rat model [15]. The presence of GLP-1 receptors in both the central and peripheral nervous systems has been demonstrated through animal research [19].

GLP-1(7-36) NH2, an amidated peptide consisting of 30 amino acids, is the major form of GLP-1, which is produced by the cleavage of proglucagon in intestinal L cells [20,21]. It operates by binding to the GLP-1 receptor (GLP-1R), which belongs to the G protein-coupled receptor family. GLP-1 is known for its many metabolic functions, such as helping glucose-dependent insulin secretion [22], reducing hunger [23], slowing down the emptying of the stomach [24], stimulation of β-cell proliferation [25], increasing natriuretic and diuretic activities [26], and helping people sleep [27]. GLP-1 is also well known for its neuroprotective and cardioprotective properties, which include anti-apoptotic [28] and anti-inflammatory effects [29], and its influence on memory, learning, palatability, and reward behavior [30,31]. The advantageous impacts of GLP-1 on the central nervous system are mostly evidenced in murine models of stroke, Parkinson’s disease, Alzheimer’s disease, and amyotrophic lateral sclerosis [32,33,34]. However, there are indications that GLP-1 may also work in non-pancreatic tissues that do not have GLP-1R, indicating that the hormone may act through receptors or mechanisms that are not yet understood [35]. For instance, GLP-1R is expressed by both astrocytes and microglia [36,37]. GLP-1R activation in these glial cells has been demonstrated to have neuroprotective benefits, including lowering neuroinflammation and accelerating nerve injury recovery. This implies that focusing on GLP-1R in astrocytes and microglia may be a viable therapeutic approach for the management of neuropathic pain and neurodegenerative diseases [36,37].

Several GLP-1 receptor agonists (GLP-1RAs) are approved for managing type 2 diabetes and facilitating weight control [38]. Recent research indicates that GLP-1RAs may enhance the management of various disorders impacting the central nervous system through the reduction in microglial cell activity [39]. Numerous studies indicate that microglial cell activation is crucial in the progression of diabetic neuropathic pain (DNP) [40]. Research indicates that DNP leads to a heightened activation of spinal microglial cells in comparison to normal rats [41], and intrathecal administration of minocycline, a strong microglial cell inhibitor, attenuates allodynia and hyperalgesia in DNP rats [42]. According to a new study by Wang et al., activating spinal microglial GLP-1 receptors with GLP-1(7-36) and exenatide effectively reduced inflammatory pain, neuropathic pain from nerve damage, and bone cancer pain [43]. The specific function of spinal GLP-1R in DNP is not yet fully understood and necessitates additional investigation.

Multiple studies demonstrate that oxidative stress plays a crucial role in the development of diabetic neuropathic pain (DNP) [44]. Oxidative stress is caused by excessive free radical generation and the inadequacy of antioxidant defense mechanisms [45]. Studies have shown that elevating intracellular reactive oxygen species (ROS) levels can induce neuronal degeneration and enhance sensitivity in pain scenarios [46]. Inhibiting the generation of ROS is a more effective method for alleviating oxidative stress than merely neutralizing ROS. The Nrf2 and Keap1 signaling pathways are crucial for mitigating damage from oxidative and inflammatory stress. Nrf2 regulates cellular responses to stress by promoting the synthesis of antioxidant and detoxifying enzymes. This shields cells from the damaging effects of oxidative stress. Researchers now acknowledge GLP-1’s multifaceted functions beyond glucose regulation in numerous tissues and organs, including the brain, kidney, and heart. GLP-1 and GLP-1 receptor agonists are effective in treating a number of long-term conditions, such as diabetes, thanks to the Nrf2 signaling pathway’s antioxidative mechanisms [47].

These data suggest that GLP-1R agonists can mitigate DNP by activating spinal GLP-1R, decreasing oxidative stress, and enhancing sleep quality. To our knowledge, no preclinical investigation has reported the efficacy of the GLP-1R agonist SEMA in treating diabetic neuropathic pain. This study investigated the effectiveness of the GLP-1 agonist SEMA in reducing diabetic neuropathic pain in a streptozotocin-induced diabetic rat model.

## 2. Materials and Methods

### 2.1. Establishment of Diabetic Neuropathic Pain Model and Drug Treatment

We used a single intraperitoneal injection of freshly prepared STZ at a dosage of 60 mg/kg body weight in 0.01 M of the citrate buffer, pH 4.5, to induce diabetes. We have administered 0.01 M of the citrate buffer to the control group of animals. We have used a tail vein puncture to obtain blood following a four-week STZ injection. Diabetic rats were defined as having blood glucose levels more than 250 mg/dL.

### 2.2. Study Design and SEMA Administration

**Figure 1:**
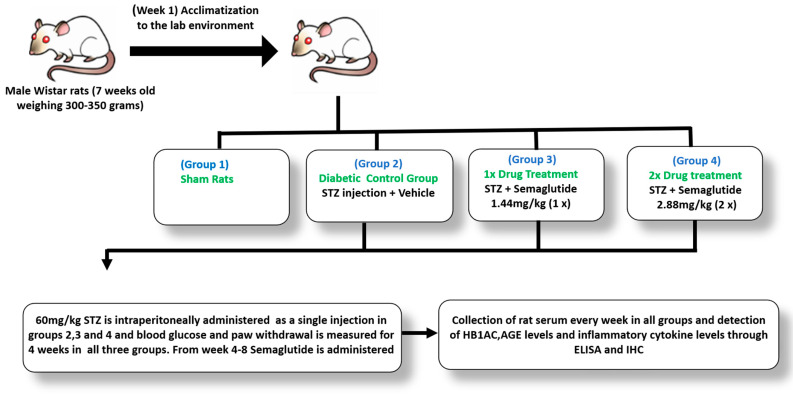
Overview of the experimental design.

As illustrated in Figure 1, we randomly categorized 40 male *Wistar* rats, weighing about 350 g, into four groups, each containing at least 6–8 animals. All the drugs were prepared in normal saline and STZ control rats were given equal volumes of saline without the drug. The drugs were administered in the following combinations orally for a duration of four weeks: DNP + saline, DNP + SEMA 1× (1.44 mg/kg), and DNP + SEMA 2× (2.88 mg/kg). Prior to drug administration, the body weights, paw withdrawal thresholds, thermal latencies, blood samples, and weight-bearing outcomes were assessed as baseline values. 14 mg SEMA tablets were sourced from Rybelsus^®^. We provided food and water to the rats, kept them in solitary cages, and exposed them to a 12 h light/dark cycle. We promptly euthanized rats exhibiting neurological disorders. Once-daily oral semaglutide with 1× and 2× doses was chosen for our study by conversion of the regular human dose to animal dosage, where 1× is equivalent to a 14 mg single oral pill in humans and 2× equates to a 28 mg dose in humans. We chose the oral formulation for our study because previous studies have shown that a 14 mg oral dosage in humans has comparable or superior efficacy and similar tolerability compared to the injectable version [48]. Furthermore, oral formulations may enhance convenience, acceptance, and adherence to GLP-1RA therapy, offering an alternative to facilitate the attainment of glycemic targets, especially in patients hesitant to commence injectable medications [49]. In addition, some recent research in type 2 diabetes patients revealed that individuals preferred oral semaglutide over injectable versions of other GLP-1 agonists [50,51].

### 2.3. Assessment of Thermal Hyperalgesia and Mechanical Allodynia

We evaluated the sensitivity of the plantar surface to tactile stimuli using an automated von Frey Dynamic Plantar Anesthesiometer (Ugo Basile, Comerio, Italy). We housed each rat in a separate plastic cage, measuring 25 cm long, 10 cm wide, and 14 cm high, with a wire mesh floor and allowed a 15 min acclimatization period before each test session. We induced a paw withdrawal response by applying an increasing force with a blunt-end metal filament (0.5 mm in diameter) at the center of the hindpaw’s plantar surface. The force started below the detection threshold, gradually increased from 1 g to 50 g in increments of 1 g over 20 s, and remained at 50 g for an additional 10 s. We defined the tactile threshold as the force required to elicit reflexive hindpaw withdrawal and recorded it as the average of three measurements taken at one-minute intervals.

We measured thermal hyperalgesia using a Hargreaves radiant heat apparatus (7371; Ugo Basile, Comerio, Italy; infrared setting 80). We housed rats in a plastic cage that measured 23 cm in length, 18 cm in width, and 14 cm in height, and allowed it a 15 min acclimatization period before the behavioral test. We placed the cage on a glass plate above the plantar test apparatus, positioning a movable noxious heat source directly beneath the hindpaw’s plantar surface. When the device was turned on, it sent a steady stream of infrared heat to the plantar surface. This caused a clear paw withdrawal reflex that stopped an automatic timer that relied on infrared reflection. We alternately tested the hindpaws, allowing a 5 min interval between consecutive tests, and averaged the latency measurements for each hindpaw across three trials in each test session. The baseline threshold ranged from 8 to 10 s in normal rats, with a cutoff time of 22 s implemented to avoid tissue damage.

Analysis of data. Thermal and mechanical responses were normalized to baseline averages and analyzed using a one-way repeated measures analysis of variance (ANOVA) with SPSS 9.0 software (SPSS, Chicago, IL, USA) and InStat 3.05 (GraphPad Software version 7.0, Inc., La Jolla, CA, USA). We conducted multiple post hoc comparisons using Least Significant Difference (LSD) tests. We employed a 95% confidence interval for all statistical comparisons, reporting the error as the standard error of the mean.

### 2.4. Biochemical Assays

After four weeks of SEMA treatment, the rats underwent overnight fasting, followed by the extraction of blood samples via the retroorbital plexus using glass capillaries. The blood was allowed to coagulate, and the serum was separated through centrifugation at 4000 rpm for 10 min. The total cholesterol, triglycerides, HDL, LDL, and VLDL were quantified using commercially available assays from Sigma Aldrich [52]. The free fatty acid (FFA) levels were measured using commercial kits purchased from Randox Laboratories (Crumlin, Co., Antrim, UK). As per Randox Laboratories’ guidelines, the biochemical tests were carried out.

### 2.5. Quantification of Advanced Glycation End Products (AGEs) Using ELISA

Four weeks post-drug treatment, on the final day, blood is collected through heart puncture and the plasma levels of AGEs is determined through the OxiSelect AGE connection ELISA kit, Cell Biolabs Inc. (San Diego, CA, USA), according to the instructions of the manufacturer.

### 2.6. Pro-Inflammatory Cytokines Measurement in Dorsal Horn Homogenates with ELISA

Six of the rats in each group (Sham, DNP-Veh, SEMA 1×, and SEMA 2×) were exsanguinated under isoflurane anesthesia for four weeks following SEMA or vehicle treatment. The dorsal quadrant portion of the lumbar spinal cord was then detached and kept at −80 °C until it was needed. Using an ultrasonic cell disruptor (Misonix, Inc., Farmingdale, NY, USA), we homogenized the spinal cord tissues in ice-cold 1× RIPA lysis buffer (Thermoscientific, Walthman, MA, USA). The homogenate was then centrifuged at 13,000 RPM for 30 min at 4 °C. Using the appropriate ELISA kits (IL-1β, IL-6, and Tumor Necrosis Factor-alpha (TNF-α): Rat IL-1β ELISA kit, Rat IL-6 ELISA kit, and Rat TNF-α ELISA kit were purchased from Invitrogen Corporation, Camarillo, CA, USA and in accordance with the manufacturer instructions, the supernatant was collected in order to measure the levels of pro-inflammatory mediators (IL-1β, IL-6, and TNF-α).

### 2.7. Immunocytochemistry and Image Analysis

The Iba1 antibody was acquired from Santa Cruz Biotechnology (1022-5) Sc-32725 (Santa Cruz, CA, USA) The GFAP antibody was acquired from Atlas antibodies (Stockholm, Sweden) with the designation HPA056030. TNF-α (NBP2-34303) and IL-1β (NB-600633) were obtained from Novus Biologicals (Littleton, CO, USA). After the completion of the experiments, the animals were sacrificed using isoflurane anesthesia (Abbott Laboratories Ltd., Queenborough, Kent, UK). The lumbar enlargement (L5–S3) of the spinal cord was promptly excised and fixed with 4% formaldehyde for 8 h, and then we fixed them with 30% sucrose for 8 h. The samples were then paraffinized and sectioned (10 µM). The slides were incubated for 12 h at 4 °C with either corresponding antibody at 1:100. We then stained the slides with DAPI (Sigma-Aldrich, St. Louis, MO, USA) for 1 h and scanned them using a Pannoramic 205 FLASH II slide scanner. We processed the scanned images using the case viewer software. We performed quantitative analyses using the ImageJ software (https://imagej.net/).

The data are presented as the mean ± standard deviation (SD). All graphical representations and statistical analyses were conducted using GraphPad Prism version 6.01. A two-way ANOVA accompanied by the Holm–Sidak multiple comparisons test was employed for statistical analysis. We considered *p*-values less than 0.05 significant.

## 3. Results

### 3.1. Oral Administration of SEMA Attenuated DNP

**Figure 2:**
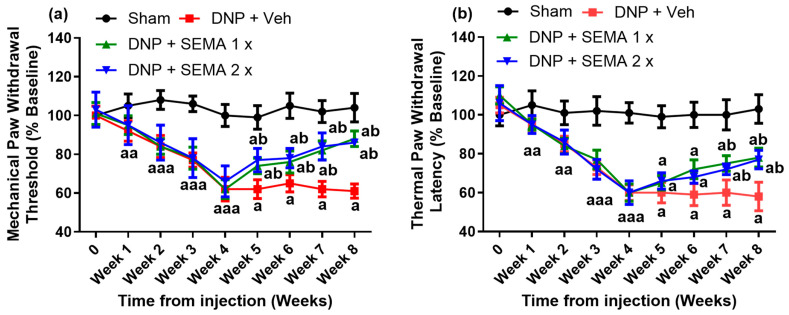
(**a**) Rats’ mean mechanical paw withdrawal threshold and (**b**) mean thermal paw withdrawal latency were normalized following a single intraperitoneal injection of streptozotocin (STZ, 60 mg/kg i.p.). The withdrawal threshold of the rats’ paws and thermal latency were assessed each week post injection *(n* = 6). Control rats received the same volume of normal saline (*n* = 6) 4 weeks after STZ injection. From week 4, SEMA was orally administered at a dose of 1× (1.44 mg/kg) or 2× (2.88 mg/kg) to rats daily for 4 weeks (week 4–8). Data are presented as mean ± SEM (*n* = 6). a *p* < 0.05 vs. Sham; b *p* < 0.05 vs. DNP group.

The mechanical paw withdrawal threshold and thermal paw withdrawal latency both went down significantly in DNP rats four weeks after an STZ injection, as shown in Figure 2a,b (red curves). On average, these decreased by about 40%. In the four weeks after drug treatment, from weeks 5 to 8, SEMA treatment greatly increased the mechanical paw withdrawal threshold and thermal paw withdrawal latency (Figure 2a,b, blue, and green curves). We observe a 25% increase in the mechanical paw withdrawal threshold and an 18% increase in the thermal paw withdrawal latency during the fourth week of SEMA therapy. The concentration of SEMA did not significantly affect the mean mechanical paw withdrawal threshold and thermal paw withdrawal latency (Figure 2a,b, blue, and green curves).

### 3.2. SEMA Partially Mitigates STZ-Induced Hyperglycemia and HbA1C Increase Without Influencing Body Weight

**Figure 3:**
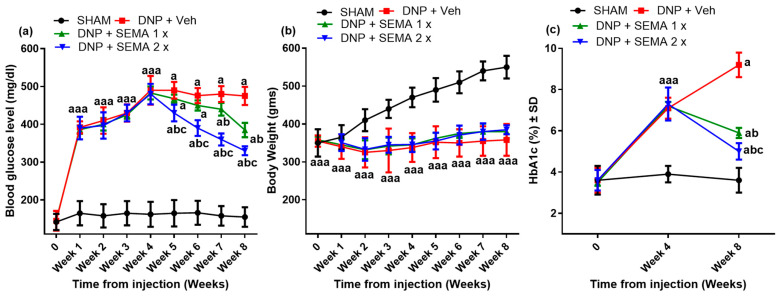
(**a**) Blood glucose concentrations (mg/dL) (**b**) Changes in body weight and (**c**) Hb1Ac levels measured at different weeks after a single injection of streptozotocin (60 mg/kg i.p). Sham rats received the same volume of normal saline (*n* = 6). 4 weeks after STZ injection, SEMA administered at a dose of 1× (1.44 mg/kg) or 2× (2.88 mg/kg) to rats daily for 4 weeks (week 4–8). Data are presented as mean ± SEM (*n* = 6). a *p* < 0.05 vs. Sham; b *p* < 0.05 vs. STZ-DNP group; c *p* < 0.05 vs. DNP + SEMA 1×.

Four weeks after the STZ injection, STZ-treated rats exhibited a significant elevation in blood glucose levels (Figure 3a, red, blue, and green curves) relative to the sham group (Figure 3a, black curve). Analogous to hyperglycemia, there was STZ-induced substantial weight loss (Figure 3b, red, blue, and green curves) at the four-week mark in comparison to the sham group (Figure 3b, black curve). Similarly, the STZ injection resulted in a spike of HbA1C (Figure 3c, red, blue, and green curves) compared to the sham group (Figure 3c, black curve). The 2× dose of SEMA was significantly more effective in glucose level reduction than the 1× dose (Figure 3a, blue curve). During weeks 6 to 8, the body weight of the SEMA-treated group was greater than that of the STZ control group, despite the fact that the difference was not statistically significant. Similar to the glycemic control, the SEMA 2× dose had a better reduction of HbA1C levels than the 1× dose (Figure 3c, blue curve).

### 3.3. SEMA Treatment Reduced Circulating AGEs Level in Diabetic Rats

**Figure 4:**
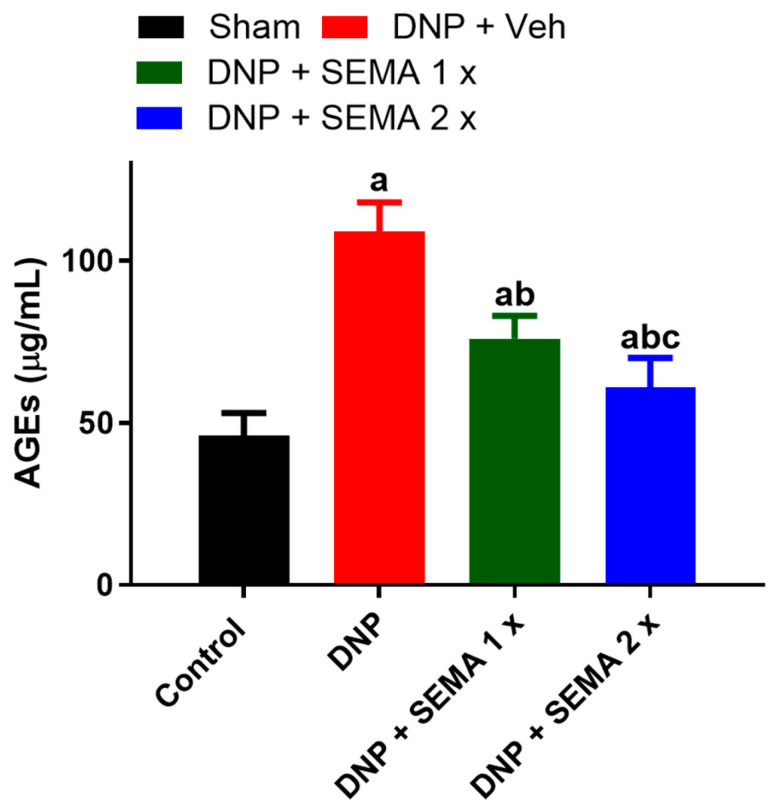
The effect of STZ-induced diabetes on plasma levels of advanced glycation end products (AGEs) in rats with STZ-induced diabetic neuropathy, assessed four weeks after SEMA administration. Data are presented as mean ± SEM (*n* = 6). a *p* < 0.05 vs. Sham; b *p* < 0.05 vs. STZ-DNP group; c *p* < 0.05 vs. DNP + SEMA 1×.

The AGEs level in the Sham group of rats was measured at 50 ± 4.5 µg/mL (Figure 4, black curve). In the STZ-treated rats, the level of AGEs was significantly elevated in comparison to the sham group (115 ± 5.5 µg/mL vs. 50 ± 4.5 µg/mL) (Figure 4, red curve). Notably, following SEMA therapy, the levels of AGEs were considerably (*p* < 0.05) decreased in a dose-dependent manner (75 ± 4.8 µg/mL vs. 70 ± 4.9 µg/mL) (Figure 4, green and blue curves).

### 3.4. SEMA Reduced the Expression of Inflammatory Marker Levels in the Spinal Dorsal Horn

**Figure 5:**
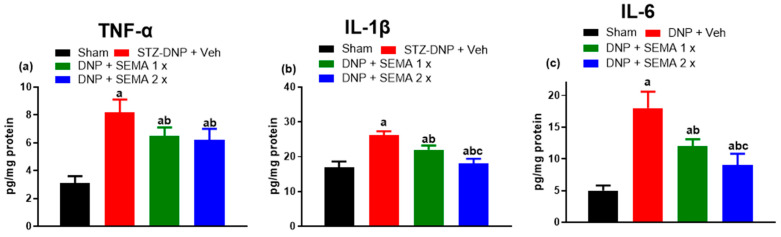
SEMA’s effects on the levels of (**a**) TNF-α, (**b**) IL-1β, and (**c**) IL-6, in the spinal dorsal horn of the DNP rats. The spinal dorsal horn’s IL-1β, TNF- α, and IL-6 levels were measured using ELISA. Data are presented as mean ± SEM (*n* = 6). a *p* < 0.05 vs. Sham; b *p* < 0.05 vs. STZ-DNP group; c *p* < 0.05 vs. DNP + SEMA 1×.

The levels of molecular inflammatory markers were evaluated in the spinal dorsal horn among various treatment groups. Figure 5 presents the results. The levels of all three inflammatory markers significantly rose after four weeks of STZ treatment (Figure 5a–c, red bar). The levels of TNF-α, IL-1β, and IL-6 were significantly lower in SEMA-treated diabetic animals compared to those in the STZ group (Figure 5a–c, green and blue bars). The reduction was in a dose-dependent manner for the inflammatory cytokines IL-1β and IL6, with the 2× dose showing a superior reduction than the 1× dose. However, there is no significant difference in TNF-α levels between the 1× and 2× doses.

### 3.5. SEMA Improves Lipid Profile

Table 1 indicates that following the induction of diabetes in rats, there was a significant decrease in HDL levels (*p* < 0.05) and an increase in TC, TG, VLDL, LDL and FFA levels (*p* < 0.05, respectively) compared to the control group. The results presented in Table 1 demonstrate that the administration of SEMA at dosages of 1× and 2× to diabetic rats over a four-week period led to a significant reduction in LDL (*p* < 0.05) and cholesterol (*p* < 0.05) levels when compared to the diabetic control group. Treatment with both 1× and 2× SEMA did not influence HDL levels. SEMA administration significantly decreased VLDL, LDA, and FFA levels in diabetic rats at doses of 1× (*p* < 0.05) and 2× (*p* < 0.05) compared to the diabetic cohort.

### 3.6. SEMA Suppresses Microglial and Astrocyte Activation in the Spinal Cord of DNP Rats

**Figure 6:**
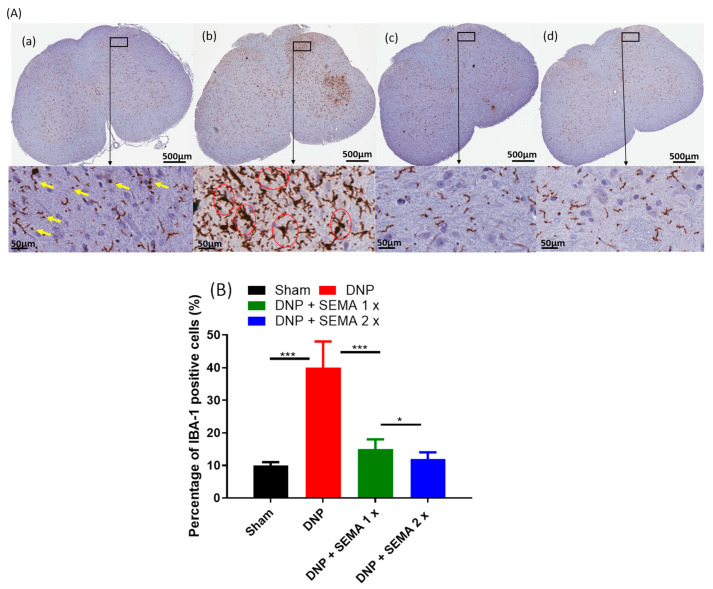
(**A**) Impact of SEMA treatment on microglial cell expression in the spinal cords of the DNP rats. Four weeks post-drug therapy, spinal cord slices were fixed and labeled with the IBA-1 microglial cell marker, followed by imaging via fluorescence microscopy. The sections were obtained from (**a**) sham rats, (**b**) DNP rats (**c**) DNP + SEMA 1× and (**d**) DNP + SEMA 2×. (**B**) The quantitative analysis of activated microglial cells is conducted. The yellow arrows in the enlarged portion of figure (**a**) indicate microglia displaying a resting ramified phenotype, characterized by a small cell body with fewer than one branching process. The red circles in the magnified region of figure (**b**) indicate microglia displaying an amoeboid morphology, defined by a prominent cell body and multiple branching processes. An asterisk in figure (**B**) indicates a statistically significant difference among sham versus DNP + Veh, DNP + Veh versus DNP + SEMA 1.44 mg/kg, and DNP + SEMA 1.44 mg/kg versus DNP + SEMA 2.88 mg/kg * *p* < 0.05; *** *p* < 0.001, (*n* = 6 animals per group).

**Figure 7:**
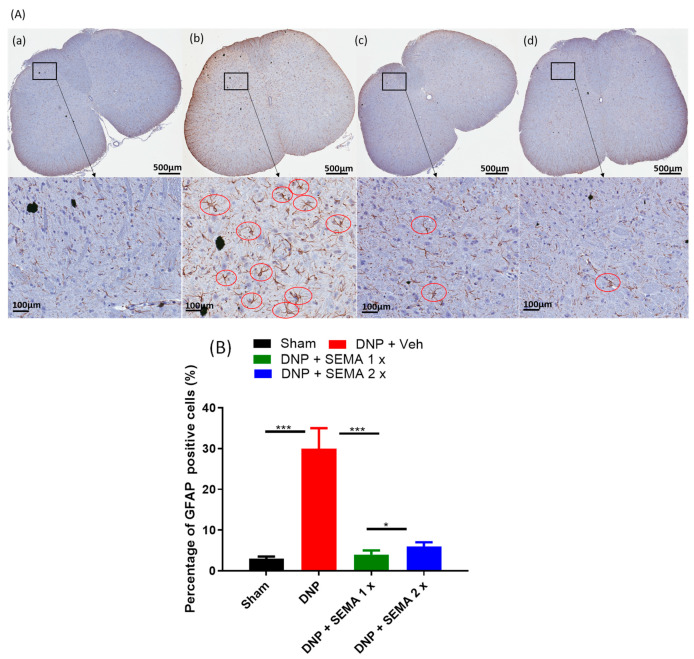
(**A**) The effect of SEMA treatment on astroglial GFAP staining in the spinal cords of the DNP rats. Four weeks post-drug therapy, spinal cord slices were preserved, labeled with the GFAP marker, and analyzed via fluorescence microscopy. The sections were obtained from (**a**) sham rats, (**b**) DNP rats (**c**) DNP + SEMA 1× and (**d**) DNP + SEMA 2×. (**B**) The quantitative analysis of activated astroglial cells is presented. STZ rats exhibited GFAP-positive cells characterized by the typical stellate morphology of astrocytes, featuring numerous elaborate processes highlighted in red circles. Both 1× and 2× reduced astrogliosis. An asterisk indicates a statistically significant difference among sham versus DNP + Veh, DNP + Veh versus DNP + SEMA 1.44 mg/kg, and DNP + SEMA 1.44 mg/kg versus DNP + SEMA 2.88 mg/kg * *p <* 0.05; *** *p <* 0.001, (*n* = 6 animals per group).

To examine the inhibitory effect of SEMA on the DNP-induced activation of microglial cells and astrocytes, we stained the spinal cord using the microglial cell-specific marker IBA-1 and the astrocyte-specific marker GFAP (Figure 6 and Figure 7). The diabetes group significantly increased the expression levels of IBA-1 and GFAP in the dorsal and ventral horns after eight weeks of STZ injection compared to the sham group. The morphology of microglia and astrocytes in the sham group displayed small dot-like formations, indicating the resting state of microglia (Figure 6A and Figure 7A).

Following the establishment of DNP, microglia exhibited hypertrophy, while astrocytes showed somatic and dendritic hypertrophy, accompanied by processes of elongation, in comparison to sham rats (Figure 6B and Figure 7B). Treatment with SEMA 1× and 2× Figure 6(Ac,Ad) and Figure 7(Ac,Ad) significantly decreased the activation of spinal microglia and astrocytes (Figure 6B, green and brown bars) and (Figure 7B, green and brown bars). The results show that the spinal dorsal horn of the DNP rats activated microglia and astrocytes, but SEMA therapy markedly reduced this activation by correcting the pathological alterations.

### 3.7. SEMA Inhibits the Production of TNF-α and IL-1β in the Dorsal Horn of DNP Rats

**Figure 8:**
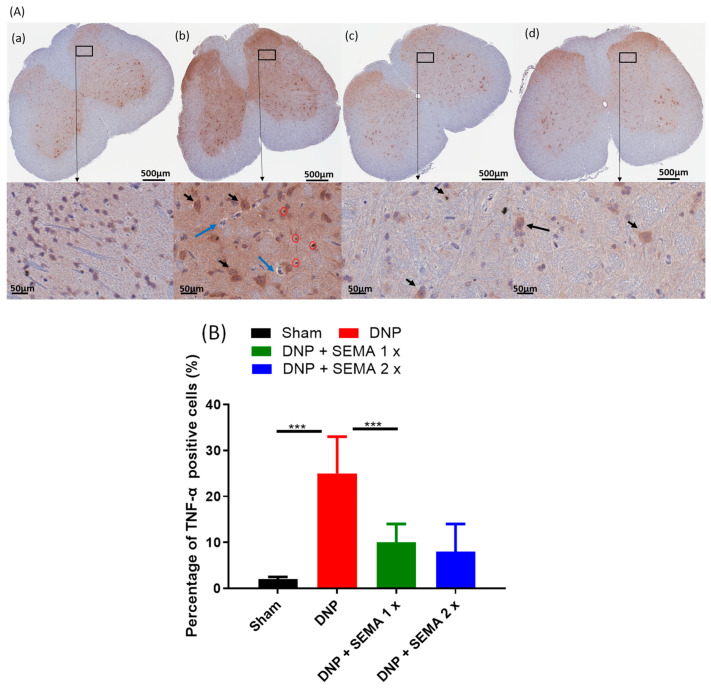
(**A**) The effect of SEMA treatment on TNF-α expression in the spinal cords of the DNP rats. Four weeks post-drug therapy, spinal cord slices were preserved, labeled with the TNF-α marker, and analyzed via fluorescence microscopy. The sections were obtained from (**a**) sham rats, (**b**) DNP rats (**c**) DNP + SEMA 1× and (**d**) DNP + SEMA 2×. (**B**) The quantitative analysis of TNF-α-positive cells. TNF-α is produced by various cell types within the spinal cord. (1) Large pyramidal cells, similar to neurons, are indicated by black arrows; (2) solitary cells resembling lymphocytes are highlighted with red circles; and (3) clusters of inflammatory cells are marked by blue arrows. An asterisk indicates a statistically significant difference among sham versus DNP + Veh, DNP + Veh versus DNP + SEMA 1.44 mg/kg, and DNP + SEMA 1.44 mg/kg versus DNP + SEMA 2.88 mg/kg *** *p <* 0.001, (*n* = 6 animals per group).

**Figure 9:**
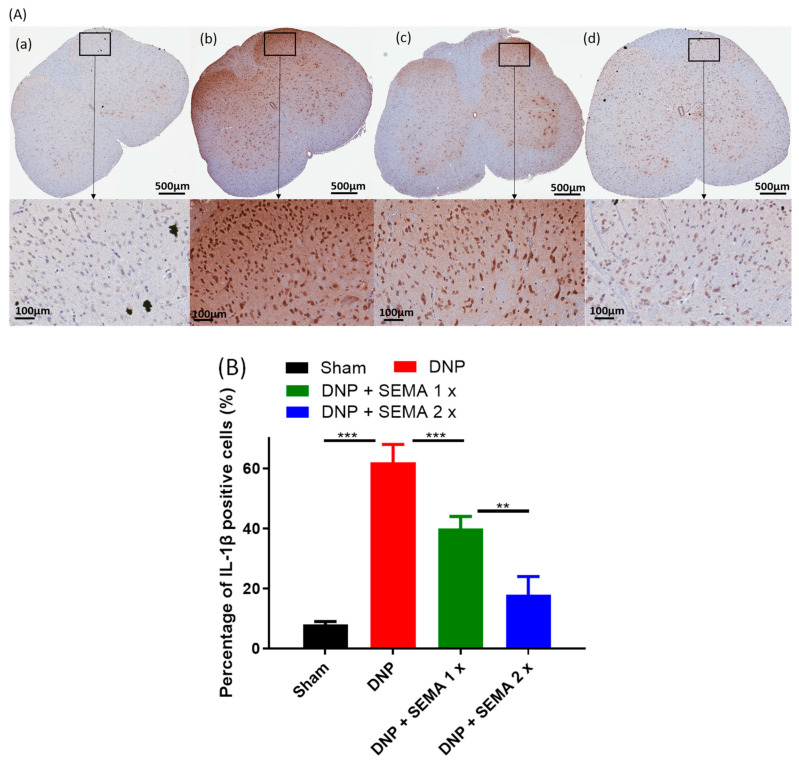
(**A**) Impact of SEMA treatment on IL-1β expression in the spinal cords of the DNP rats. Four weeks post-drug therapy, spinal cord slices were preserved and labeled with the IL-1β marker, followed by image acquisition via fluorescence microscopy. The sections were obtained from (**a**) sham rats, (**b**) DNP rats (**c**) DNP + SEMA 1× and (**d**) DNP + SEMA 2×. (**B**) The quantitative analysis of IL-1β-positive cells. In the DNP rats, a significant increase in IL-1β levels in the spinal cord was noted in comparison to sham animals. The administration of SEMA 1× resulted in a slight reduction of IL-1β expression, while animals injected with DNP and SEMA 2× exhibited staining comparable to that of sham animals. An asterisk indicates a statistically significant difference among sham versus DNP + Veh, DNP + Veh versus DNP + SEMA 1.44 mg/kg, and DNP + SEMA 1.44 mg/kg versus DNP + SEMA 2.88 mg/kg. ** *p* < 0.01; *** *p* < 0.001, (*n* = 6 animals per group).

Through immunohistochemistry, it was proven that SEMA does affect the production of the pro-inflammatory cytokines TNF-α (Figure 8A) and IL-1β (Figure 9A). There are a lot more TNF-α and IL-1β expression in the DNP rats (Figure 8B and Figure 9B, red bars) than in the sham rats (Figure 8B and Figure 9B, black bars). However, treatment with SEMA attenuated the expression of both the pro-inflammatory markers after 4 weeks at both the tested doses (Figure 8B and Figure 9B, blue and brown bars). The results demonstrate that SEMA inhibits the production of pro-inflammatory cytokines in DNP rats.

## 4. Discussion

The findings of this study demonstrated that oral SEMA reduced both mechanical allodynia and thermal hyperalgesia. Additionally, we observed an increase in the expression of microglia and astrocytes in the dorsal and ventral horns of STZ rats compared to the control and SEMA-treated rats. Studies show that both the dorsal and ventral horns of animals that were given STZ to make them diabetic have a lot more microglial activation [53]. The activation is associated with the initiation of neuropathic pain and involves the release of pro-inflammatory cytokines. SEMA demonstrated efficacy in lowering blood glucose and serum HbA1c levels. Furthermore, SEMA was shown to lower the levels of the pro-inflammatory cytokines IL-1β, IL-6, and TNF-α in the blood as well as the levels of IL-1β and TNF-α in the dorsal horn.

Oxidative stress induces multiple inflammatory mediators that contribute to lipid membrane destruction and tissue injury, ultimately resulting in the development of DNP [54]. Our study demonstrates that STZ-induced DNP led to a significant increase in the serum levels of all three pro-inflammatory cytokines TNF-α, IL-1β and IL-6, and our findings align with prior research indicating that uncontrolled hyperglycemia triggers a series of inflammatory responses in STZ-DNP rats [55]. In addition to their antioxidant properties, GLP-1 agonists have been identified as neuroprotective agents by modulating inflammatory responses via inhibition of the nuclear factor kappa-light-chain-enhancer of activated B cells (NF-κB) pathway [35]. A prior pre-clinical investigation demonstrated that the GLP-1 receptor agonist vildagliptin directly inhibited NF-κB and subsequent pro-inflammatory cytokine cascades in a rotenone rat model of Parkinson’s disease [56]. Treatment with SEMA led to a significant reduction in TNF-α, IL-1β and IL-6. Our findings align with prior research indicating that SEMA effectively addresses peripheral neurodegenerative diseases via its anti-inflammatory and antioxidant properties [57]. Additionally, we found that SEMA positively influences the lipid profile by decreasing the levels of TC, TG, VLDL, LDL, and FFA in diabetic rats. Altogether, our findings show that GLP-1RA SEMA lowers DNP. This is most likely achieved by stopping the activation of spinal microglia and astrocytes and lowering levels of pro-inflammatory cytokines in the blood.

Activated microglia and astrocytes have distinct functional properties and induce the release of diverse inflammatory cytokines in reaction to pathogenic occurrences in the central nervous system and are known to play a critical role in motor neuron degeneration [58,59,60]. Microglia and astrocytes exhibit significant stimulation in DNP [44]. A recent study indicates that cytokine production in the central nervous system is associated with the activation of microglia and astrocytes [40,53,61]. GLP-1 signaling appears to positively affect various factors related to the pathophysiology of neurodegenerative diseases, such as ER stress, disrupted redox homeostasis, autophagy, and chronic inflammation. Consequently, GLP-1 receptor agonists have emerged as potential therapeutic options for reducing neuronal loss and neuroinflammation [62]. Recent findings demonstrate that GLP-1 receptor agonists (GLP-1RAs) promote neurogenesis, improve neuronal survival and synaptogenesis in animal models of neuronal injury, alleviate neuroinflammation, and/or decrease pathogenic markers of protein aggregation [63]. In animal models of chemical parkinsonism, treatment with GLP-1RA liraglutide or exenatide-4 demonstrated the ability to enhance neurogenesis, safeguard dopaminergic neurons and dopamine production in the substantia nigra, and aid in the recovery of motor deficits [64,65,66,67]. The research demonstrates a correlation between advanced glycation end products (AGEs) produced by hyperglycemia and the occurrence of diabetic neuropathy [68]. AGE-modified peripheral nerve myelin is prone to macrophage phagocytosis, leading to segmental demyelination. [68]. Advanced glycation end-products (AGE) induce axonal shrinkage and degeneration, along with compromised axonal transport and alteration of key axonal cytoskeletal proteins, such as *tubulin*, *neurofilament*, and *actin* [68]. Furthermore, glycation of the extracellular matrix protein laminin impairs regenerative capabilities in diabetic neuropathy. Recent findings indicate that advanced glycation end products (AGEs) and their receptors (RAGE) are localized together in diabetic peripheral nerves [68]. AGEs and AGE/RAGE interactions result in oxidative stress in diabetic neuropathy, activate microglia, and increase pro-inflammatory cytokine levels, which contribute to neurological dysfunction, including impaired pain perception [68].

The present study indicates that DNP may increase AGEs, potentially activating microglia and astrocytes, which in turn results in the production of pro-inflammatory cytokines. SEMA treatment reduced the DNP-induced increase in AGEs and inhibited the activation of microglia and astrocytes. In our rats, semaglutide administration did not yield weight reduction. This difference may arise from the majority of the research involving type 1 diabetes patients being conducted on overweight or obese individuals, whereas our rats were lean. Nevertheless, other previously approved GLP-1R agonists for weight loss treatment like Liraglutide did not affect body weight in STZ rats similar to our study [69,70].

These data indicate that SEMA treatment may be effective in reducing DNP-induced inflammation by inhibiting the activation of microglial cells and astrocytes. The activation of microglia and the secretion of pro-inflammatory cytokines can result in the infiltration of immune cells, such as macrophages and lymphocytes, at the site of spinal cord injury [71], one of the primary attributes of inflammation within the innate immune response [71]. Our findings demonstrate that SEMA may substantially reduce DNP-induced activation of microglia and astrocytes in the spinal cord dorsal horn, indicating its potential clinical utility in DNP prophylaxis.

## 5. Conclusions

The findings of our research demonstrate that SEMA significantly alleviates DNP-induced mechanical allodynia and thermal hyperalgesia. Furthermore, SEMA decreased the activation of microglia and astrocytic cells in the spinal dorsal horn of DNP rats. It also reduced the concentrations of pro-inflammatory cytokines in the spinal dorsal horn. Our findings establish the groundwork for the potential therapeutic use of SEMA in the effective management of DNP and other chronic pain modalities.

## Figures and Tables

**Table 1:** Impact of SEMA treatment over four weeks on lipid profile and free fatty acids in STZ diabetic rats. Results are presented as mean ± SEM. Significant differences exist in comparison to the STZ group. a *p* < 0.05 vs. Sham; b *p* < 0.05 vs. DNP group; c *p* < 0.05 vs. DNP + SEMA 1×.

Groups	Total Cholesterol (mg/dL)	Triglycerides (mg/dL)	HDL (mg/dL)	VLDL (mg/dL)	LDL (mg/dL)	FFA (mmol/L)
Control	56.4 ± 1.58	93.2 ± 4.24	57.1 ± 5.16	19.2 ± 3	2.1 ± 0.1	0.60 ± 0.12
DNP + Veh	68.8 ± 1.25 ^a^	131.1 ± 10.3 ^a^	44 ± 1.51 ^a^	27.1 ± 1.9 ^a^	9.4 ± 1.53 ^a^	1.25 ± 0.08 ^a^
DNP + SEMA 1×	57.4 ± 1.16 ^ab^	109 ± 3.11 ^ab^	45 ± 1.11 ^ab^	18.9 ± 2.5 ^ab^	5.7 ± 1.12 ^ab^	0.71 ± 0.04 ^ab^
DNP + SEMA 2×	52.3 ± 2.1 ^abc^	104 ± 5.18 ^abc^	40 ± 3.41	15.7 ± 3.4 ^abc^	5.9 ± 1.41 ^ab^	0.68 ± 0.03 ^ab^

## Data Availability

The data presented in this study are available within the article.
